# The potential mechanisms underlying phthalate-induced hypospadias: a systematic review of rodent model studies

**DOI:** 10.3389/fendo.2024.1490011

**Published:** 2024-12-04

**Authors:** Youtian Zhang, Jian Wang, Hongchao Yang, Yong Guan

**Affiliations:** ^1^ Department of Urology, Tianjin Children’s Hospital/Tianjin University Children’s Hospital, Tianjin, China; ^2^ Department of Pediatric Surgery, Qilu Hospital of Shandong University Dezhou Hospital (Dezhou People’s Hospital), Dezhou, Shandong, China

**Keywords:** phthalates, hypospadias, molecular mechanisms, sex steroids, ROS

## Abstract

**Objectives:**

Maternal exposure to environmental endocrine disruptors, such as phthalates, during pregnancy is a significant risk factor for the development of hypospadias. By consolidating existing research on the mechanisms by which phthalates induce hypospadias in rodent models, this systematic review aims to organize and analyze the discovered mechanisms and their potential connections.

**Methods:**

The study involved all articles that explored the mechanisms of phthalate-induced hypospadias using rodent models. A comprehensive search of the PubMed and Web of Science databases was conducted using the terms “hypospadias” and “phthalates” before January 20, 2024. Then, two investigators screened for studies worthy of inclusion by setting inclusion and exclusion criteria.

**Results:**

Of the initial 326 search results, 22 were included in the subsequent analysis. Based on the commonalities among different results, the mechanisms of phthalate-induced hypospadias could be categorized into the following five groups: sex steroids-related signaling pathways (n=10), epithelial-mesenchymal transition (n=6), autophagy (n=5), apoptosis (n=4) and angiogenesis (n=2). Among these, sex steroids-related signaling pathways might serve as a central regulator among all mechanisms, and reactive oxygen species (ROS) also played an important mediating role.

**Conclusion:**

The systematic review indicates that phthalates may initially disrupt the balance of sex steroids-related pathways, leading to abnormally elevated levels of ROS and subsequently to other functional abnormalities, ultimately resulting in the development of hypospadias. All these findings will help to improve prevention strategies during pregnancy to reduce the adverse effects of phthalates on the offspring.

## 1 Introduction

Hypospadias ranks as one of the most common congenital malformations in the male genitourinary system, exhibiting a global incidence rate ranging from 0.6 to 34.2 cases per 10,000 live births (1). It is a male birth defect which is characterized by the ectopic urethral opening being displaced along the ventral side of the penis. Although surgical correction is possible, the procedure and subsequent post-operative complications undeniably present significant challenges for affected children. Therefore, elucidating the etiology of hypospadias has become an urgent scientific challenge, as it enables the development of potential preventive strategies. Current perspectives suggest that the pathogenesis of hypospadias is multifactorial, encompassing genetic factors, maternal influences, and environmental determinants (2). However, empirical genome-wide association studies (GWASs) on hypospadias in human cohorts indicate that detectable genetic mutations account for less than 10% of the observed cases (3, 4), suggesting that non-genetic factors may play a more significant role in the development of this congenital malformation. Consequently, the speculation that excessive maternal intake of environmental endocrine-disrupting chemicals (EDCs) leads to abnormal embryonic development of fetal genitalia has garnered increasing attention.

The development of male external genitalia is governed by a precise signaling pathway transduction within the genital tubercle (GT) of the embryo, guided by hormonal cues. This process orchestrates the orderly differentiation, migration, and proliferation of cells, resulting in the formation of a tubular urethra and a fully developed foreskin (5, 6). During the early hormone-independent stage of genital development, the urethral plate gradually forms along the embryonic midline. Subsequently, in the hormone-dependent phase, the influence of sex steroids-related pathways on external genitalia development becomes increasingly significant. A balanced signaling of androgens and estrogens jointly supports the normal masculinization process of the GT, leading to the elongation of the urethra, which ultimately encapsulates and forms the structures of the male external genitalia (7, 8). The WNT, FGF, BMP, and Hedgehog (HH) signaling pathways play crucial roles in the development and patterning of various tissues within the penis (9, 10). These pathways are interconnected and exhibit significant crosstalk within their genetic networks. Under the regulation of estrogens and androgens, they collaboratively facilitate signaling between the epithelium and mesenchyme, coordinating the spatiotemporal development of the external genitalia (11).

EDCs are a broad class of exogenous agents known to interfere with the endocrine system by imitating, blocking, or otherwise modifying hormonal signals, with a pronounced impact on sex steroids (12). Several studies have indicated that pregnant women’s exposure to high levels of EDCs may disrupt critical signaling pathways responsible for sexual differentiation in fetal development, potentially leading to genitourinary malformations, such as hypospadias (13, 14). Phthalates, serving as plasticizers, rank among the most frequently encountered EDCs in the daily lives of pregnant women due to their extensive use in a wide array of consumer and industrial products, including food packaging, toys, medical devices, and personal care items (15–17). These compounds can easily leach into the environment due to their non-covalent bond with plastics. Subsequently, when absorbed by pregnant women through pathways such as ingestion, inhalation, and dermal absorption, these substances can cross the placental barrier and ultimately impact fetal embryonic development (18, 19).

The positive correlation between prenatal exposure to phthalates and the incidence of hypospadias in offspring has been confirmed in epidemiological studies, highlighting the necessity for experimental investigation of the specific mechanisms by which phthalates induce hypospadias (20, 21). Given the considerable technical and ethical challenges associated with direct research on human penile embryonic development, researchers have predominantly relied on rodent models to investigate the etiology of hypospadias. This approach typically involves administering phthalates to pregnant rodents via oral gavage to induce hypospadias phenotypes in their offspring. While a substantial body of research has identified numerous potential pathogenic mechanisms triggered by phthalates, the findings are disparate and lack a cohesive framework. This divergence largely stems from variations in experimental designs and foci across studies, which complicates the task of drawing comprehensive conclusions.

The present systematic review aims to consolidate existing research on the mechanisms by which phthalates induce hypospadias in rodent models. By categorizing identified mechanisms and examining their interrelations, this review seeks to uncover underlying core mechanisms that may govern the wide array of signaling pathways implicated in phthalate-induced hypospadias, thereby offering new insights into potential preventive strategies for this congenital condition associated with environmental endocrine disruptors.

## 2 Methods

To systematically investigate the mechanisms of phthalate-induced hypospadias, we retrieved, screened, categorized and analyzed the data according to the Preferred Reporting Items for Systematic reviews and Meta-Analyses (PRISMA) statement (22).

### 2.1 Information search

Prior to undertaking the systematic review, both investigators first identified “phthalates” and “hypospadias” as search terms. A comprehensive search was conducted in PubMed and Web of Science databases on January 20, 2024 (The specific search strategy is detailed in **Supplementary Table S1**). This was not time-limited, but the search was restricted to English-language publications. Search results were independently evaluated by two investigators for relevance to the purpose of the study.

### 2.2 Inclusion and exclusion criteria

Experimental studies on the utilization of phthalates to create animal models of hypospadias for mechanism analysis were eligible for inclusion. The exclusion criteria were 1) narrative or systematic reviews and meta-analyses; 2) epidemiological research (e.g., case-control, prospective cohort studies); 3) reports or other non-experimental studies; 4) published abstracts without full text available or publications in languages other than English; 5) other animal studies not focused on hypospadias models. Three investigators individually screened all studies to confirm eligibility. Any disagreements were resolved through discussion to optimize the screening protocol and reach consensus.

### 2.3 Data extraction

Variables for study characteristics, methodology/design and outcomes were main elements of data integration. Study characteristics variables included first author, year of publication and study subject. The following data were extracted as methodology variables: phthalate selection and treatment, *in vivo* and/or *in vitro* experimental design. Outcome variables recorded the incidence and phenotype of hypospadias in the offspring, expression of relevant pathway components, alterations in biological function and key conclusions. Two investigators independently performed the data collection process described above, and if differential results were presented, they were validated and adjudicated by a third investigator.

## 3 Results

**Figure 1:**
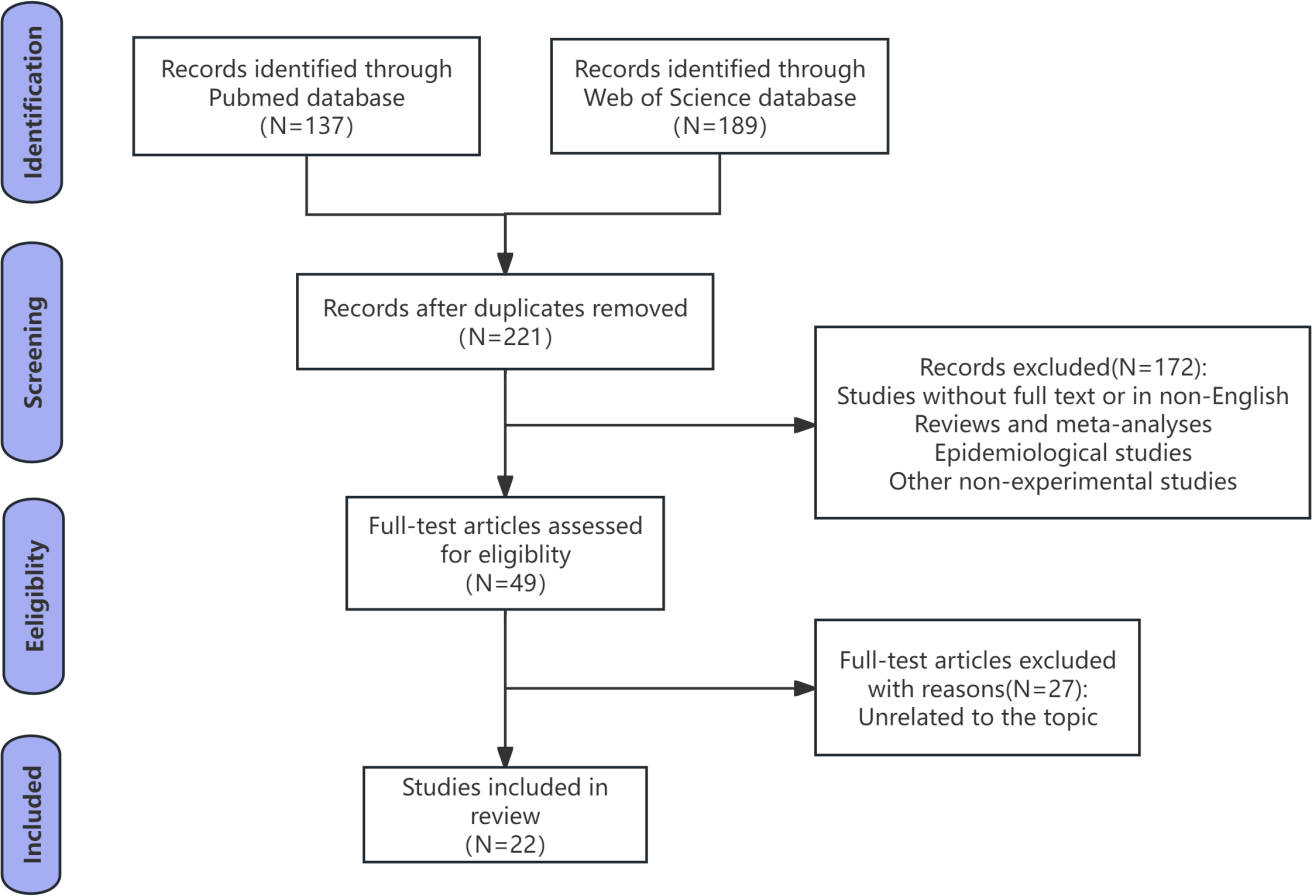
Flow diagram of search strategy.

Our search ultimately yielded 326 preliminary results, of which 105 were duplicates. The eligibility criteria and the specific process for study selection are described in **Figure 1**. Afterward, we eliminated animal model studies without full text or those not in English, as well as certain types of publications (reviews, meta-analyses, epidemiological studies, and other non-experimental studies). Through full-text screening, 27 articles unrelated to the topic of our review were further excluded, resulting in 22 papers deemed qualified for integrated analysis.

### 3.1 Study characteristics

**Table 1** summarizes the methodological designs for constructing hypospadias rodent models and the characteristics of the hypospadias offspring in the included studies. In the 22 studies reviewed, researchers used either di-n-butyl phthalate (DBP) or di-(2-ethylhexyl) phthalate (DEHP) for prenatal exposure. DBP was predominantly studied (N=16; all rat models), with 6 studies on DEHP (4 rat models and 2 mouse models). Among all rodent models, Sprague-Dawley (SD) rats were used in 20 studies, C57BL/6 mice were used in one study (23), and one study (24) did not specify the mouse strain. Due to the presence of specific urethral developmental windows, phthalate treatments were administered during days 10-19 of gestation in all studies, but the duration of exposure varied. In addition, the phthalate concentrations adopted in the experiments ranged from 100 mg/kg body weight (bw)/day to 1000 mg/kg bw/day. By comparing the incidence of hypospadias in the offspring, we could broadly see that higher phthalate concentrations were associated with greater induction success (23, 25–27). At the same time, there were also differences in the timing of offspring sample collection. After harvesting the offspring, 19 studies have directly or indirectly documented urethral developmental abnormalities in pups (23–41), which were important criteria for screening hypospadias fetuses, mainly characterized by an ectopic urethral meatus, and ventral foreskin deficiency with incomplete fusion in the middle of the skin folds.

**Table 1:** Characteristics of rodent model experiments included in the study.

Author, publication year	Animal, phthalates treatment	Sampling time	*In vitro* study subjects	Incidence of hypospadias	Characterization of hypospadias in offspring
Liu et al., 2008 (23)	C57BL/6 mice, 100/200/500mg/kg bw/day DEHP during GD12–GD17	GD19	N/A	7.1% (DEHP_100_) 14.0% (DEHP_200_) 75.7% (DEHP_500_)	The urethral opening was on the distal penile shaft and the anterior urethra became shorter and straighter, with abnormalities in the closure of the urethral seam and delay in preputial development
Zhu et al., 2009 (28)	SD rats, 750mg/kg bw/day DBP during GD14–GD18	PND 7	N/A	46.7%	The urethral orifice was opening ventrally with wider separation of the prepuce
Kim et al., 2010 25)	SD rats, 250/500/700mg/kg bw/day DBP during GD10–GD19	PND 31	N/A	0% (DBP_250_, DBP_500)_ 47.3% (DBP_700_)	The urethral opening was at the middle and proximal penile shaft with penile curvature
Zhang et al., 2011 (29)	SD rats, 750mg/kg bw/day DBP during GD14–GD18	GD19	N/A	41.3%	The urethral meatus was located ventral to the base of the external genitalia, with a wider foreskin separation
Liu et al., 2012 (30)	SD rats, 750mg/kg bw/day DBP during GD14–GD18	PND 7	N/A	43.6%	A distinct abnormal urethral opening, located ventrally, at the base of the external genitalia
Jiang et al., 2016 (31)	SD rats, 750mg/kg bw/day DBP during GD14–GD18	PND 8	N/A	42.9%	The urethra opened ventrally along the GT shaft and the ventral tissue of the urethral plate was discontinuous
Zhu et al., 2016 (32)	SD rats, 850mg/kg bw/day DBP during GD11–GD15	PND 2	N/A	N/A	A urethra opened ventrally at the base of the external genitalia
Li et al., 2017 (33)	SD rats, 750mg/kg bw/day DBP during GD13–GD18	GD19	N/A	43.6%	The urethral opening was on the ventral surface of the penis and a visible cleft was on the ventral prepuce
Zhao et al., 2018 (34)	SD rats, 750mg/kg bw/day DBP during GD14–GD18	PND 7	PUECs culture	42.3%	The urethral opened ventrally at the base of the external genitalia
Zhao et al., 2018 (44)	SD rats, 750mg/kg bw/day DBP during GD14–GD18	GD19	GT fibroblast culture	43.5%	N/A
Zhao et al., 2019 (42)	SD rats, 750mg/kg bw/day DBP during GD14–GD18	PND 7	PUECs culture	N/A	N/A
Zhou et al., 2020 (27)	SD rats, 500/750mg/kg bw/day DEHP during GD12–GD18	GD 19	Penis culture	27.6% (DEHP_500_) 36.2% (DEHP_750_)	A deepening of the ventral urethral groove and ventral shift of the urethral meatus observed by electron microscopy
Zhu et al., 2020 (43)	SD rats, 750mg/kg bw/day DBP during GD14–GD18	PND 1	PUECs culture	N/A	N/A
Tian et al., 2020 (24)	Mice (unknown), 800mg/kg bw/day DEHP during GD12–GD19	GD 19	PUECs culture	N/A	Dysgenesis of the ventral prepuce, no fissure formed in the middle
Feng et al., 2021 (35)	SD rats, 800mg/kg bw/day DBP during GD12–GD17	GD 18	GT fibroblats culture	48.8%	The urethral opening was on the body of the genital tubercle and the urogenital folds were not well fused
Xiang et al., 2021 (36)	SD rats, 750mg/kg bw/day DEHP during GD12–GD19	GD 19	N/A	41.5%	The skin folds did not merge completely in the middle and completely cover the corpus cavernosum
Zhou et al., 2022 (37)	SD rats, 750mg/kg bw/day DBP during GD14–GD18	PND 10	N/A	N/A	An ectopic urinary meatus on the penile ventral aspect
Zhou et al., 2022 (38)	SD rats, 800mg/kg bw/day DBP during GD14–GD18	PND 70	N/A	N/A	The urethral meatus ectopically opened on the penile ventral aspect. The prepuce on the ventral side was absent, while the dorsal prepuce appeared as a dorsal hood. Ventral curvature of the penis and reduction in penis size
Hua et al., 2023 (39)	SD rats, 750mg/kg bw/day DBP during GD14–GD18	PND 7	HUVECs culture Urothelial cells culture	41.8%	The urethral opened ventrally at the base of the external genitalia with wider separation of the prepuce
Zhu et al., 2023 (40)	SD rats, 500mg/kg bw/day DEHP during GD14–GD18	GD19	PUECs culture	N/A	The urethral opening was located at the junction of the penis and perineum or on the ventral side of the penis
Wu et al., 2024 (41)	SD rats, 750mg/kg bw/day DBP during GD14–GD18	PND 7	HUVECs culture	N/A	The urethra opened ventrally at the base of the external genitalia with wider separation of the prepuce and the size of the penis was noticeably reduced
Shi et al., 2024 (26)	SDrats, 500/750/1000mg/kg bw/day DEHP during GD12–GD19	PND 2	N/A	57.0% (DEHP_500_) 81.4% (DEHP_750_, DEHP_1000_)	The urethral meatus ectopically opened on the penile ventral aspect and the preputial folds did not cover the corpus cavernosum completely

In all recorded animal experiments, the day on which a vaginal plug was first observed was designated as gestational day (GD) 0, and the day of birth was considered postnatal day (PND) 1. N/A indicates that the content was not mentioned or does not apply in the included study.

To further investigate the underlying molecular mechanisms, in addition to *in vivo* experiments, 10 studies also combined *in vitro* experiments, including cell cultures [urethral epithelial cells (24, 34, 39–43), fibroblasts (35, 44), endothelial cells (28, 39, 41)] and isolated penis cultures (27). In selecting reagents for *in vitro* studies, to reflect the bioactive forms of phthalates in biological systems, the DEHP treatment group used its primary metabolite, mono-(2-ethylhexyl) phthalate (MEHP). However, in some studies, DBP rather than mono-butyl phthalate (MBP) was directly applied for cell induction (34, 39, 41–43).

### 3.2 Mechanisms of phthalate-induced hypospadias

**Table 2** outlines the implications of DBP/DEHP on various signaling pathways and their terminal outcomes. After a comprehensive review of the included studies, based on the commonalities among different results, we categorized the mechanisms of phthalate-induced hypospadias into the following five groups: sex steroids-related signaling pathways, epithelial-mesenchymal transition (EMT), autophagy, apoptosis and angiogenesis.

**Table 2:** Summary of mechanisms by which phthalates induce hypospadias in the systematic review.

Study	Phthalate	Groups	Signaling pathways	Target gene/protein (hypospadias vs. control)	Significant findings (Phthalates treated)
Liu et al., 2008 (23)	DEHP	N/A	N/A	TGF-β1↑	Overexpressed TGF-β1 might be one of the key signals resulting in DEHP-induced hypospadias
Zhu et al., 2009 (28)	DBP	N/A	N/A	Shh↓, Ptch1↓, Bmp4↓, Bmp7↓, Fgf8↓, Fgf10↓, Fgfr2↓, TGF-β1↓, TGF-βrIII↓	DBP disturbed some important genes involved in the epithelial-mesenchymal interactions
Kim et al., 2010 (25)	DBP	Sex steroids- related pathways	Androgen signaling	Srd5a2↓, AR↓, Shh↓	1) The degeneration of the seminiferous epithelium in testes 2) Significantly lower serum DHT and testosterone levels 3) Downregulation of AR, Srd5a2, and Shh may play significant roles in DBP-induced disruption of androgen signaling and penile development
Zhang et al., 2011 (29)	DBP	N/A	Wnt/β-catenin	β-catenin↓, Phospho-GSK-3β↓, GSK-3β↓, NFκB↑	DBP may affect the development of GT by downregulating the Wnt/β-catenin pathway and upregulating the NF-κB pathway in fetal male rats
Liu et al., 2012 (30)	DBP	Sex steroids- related pathways	Androgen signaling	AR↓, FGF8↓	1) Significantly lower serum testosterone levels 2) The interaction of low androgen levels and the abnormal expression of FGF8 may lead to abnormal GT development
Jiang et al., 2016 (31)	DBP	Sex steroids- related pathways	Androgen signaling	Cyp11a1↓, Hsd3b↓, Scarb1↓, Star↓, Srd5a2↓, AR↓, Shh↓, Bmp4↓, Fgf8↓, Fgf10↓, Fgfr2↓	1) Severe testicular dysplasia 2) Significantly lower serum testosterone levels 3) DBP-induced inhibition of the androgen signaling pathway may suppress the expression of androgen-related genes associated with genital development
Zhu et al., 2016 (32)	DBP	Sex steroids- related pathways	Androgen signaling	Cyp11a1↓, Hsd3b↓, Scarb1↓, Star↓, Srd5a2↓, AR↓, Shh↓, Fgf10↓, Fgfr2↓, Gli2↓, Gli3↓, Bmp4↓, Wnt5a↓, Hoxa13↓, Hoxd13↓	1) Abnormalities in testicular morphology and structure 2) Significantly lower serum testosterone levels 3) Suppression of androgen signaling may lead to abnormal expression of androgen-related genes and their downstream factors
Li et al., 2017 (33)	DBP	Autophagy Apoptosis	PI3K/Akt/mTOR	p-Akt↓, p-mTOR↓, p-S6↓	1) Significantly lower serum testosterone levels 2) DBP may enhance autophagy through inhibition of the PI3K/Akt/mTOR pathway 3) DBP-induced autophagy may resist normal apoptosis
Zhao et al., 2018 (34)	DBP	Sex steroids- related pathways Autophagy EMT	Androgen signaling	AR↓	1) Impaired androgen signaling following DBP treatment contributed to promoting autophagy 2) DBP-induced autophagy enhanced EMT in urethral epithelial cells by degrading E-cadherin via the autophagy-lysosomal pathway
Zhao et al., 2018 (44)	DBP	Autophagy Apoptosis	PERK/eIF2α	p-PERK↑, p-eIF2α↑, ATF4↑	Activation of the PERK/eIF2α pathway after DBP treatment could promote autophagy and inhibit apoptosis
Zhao et al., 2019 (42)	DBP	Autophagy	Hedgehog	HhIP↑, Ptch1↓, Gli1↓	1) ROS increased in a dose-dependent manner with DBP 2) DBP enhanced autophagy via ROS-HhIP-Gli1-autophagy axis
Zhou et al., 2020 (27)	DEHP	EMT	TGF-β/Smad	TGF-β1↓, p-Smad2↓, p-Smad3↓	DBP could reduce urethral EMT by inhibiting TGF-β/Smad signaling pathway
Zhu et al., 2020 (43)	DBP	EMT	N/A	IP3R↑	1) ROS increased in a dose-dependent manner with DBP 2) DBP inhibited EMT in urethral epithelial cells by increasing IP3R expression and calcium concentration through its oxidative stress effect
Tian et al., 2020 (24)	DEHP	Apoptosis	TGF-β/Smad	miR-494↑, Nedd4L ↓, TGF-β1↑, Smad2↑, Smad3↑	1) MiR-494 could activate the TGF-β1/Smad signaling pathway by binding to Nedd4L 2) Overexpression of miR-494 may inhibit the urethral epithelial cell apoptosis process
Feng et al., 2021 (35)	DBP	Autophagy	N/A	NONRATT008453.2↑	Overexpression of NONRATT008453.2 suppressed autophagy in GT fibroblasts
Xiang et al., 2021 (36)	DEHP	Sex steroids- related pathways	Estrogen signaling	c-Fos↑	Possible complex interrelationship between estrogen signaling and c-fos transcription
Zhou et al., 2022 (37)	DBP	Sex steroids- related pathways EMT	Androgen signaling Estrogen signaling	AR ↓ ERα↑	1) Significantly lower serum testosterone level 2) DBP had high affinities for AR and ERα proteins 3) DBP may reduce EMT through downregulation of the nuclear androgen-dependent pathway and upregulation of the cytoplasmic estrogen-dependent pathway
Zhou et al., 2022 (38)	DBP	Sex steroids- related pathways Apoptosis Angiogenesis	Androgen signaling Akt/Bad/Bax/caspase-3 NOS/cGMP	p-AKT↓, Bcl-2↓, Bax↑, caspase-3↑ eNOS↓, nNOS↓	1) Significantly decreased serum testosterone levels and obvious damage of testis 2) Activation of the Akt/Bad/Bax/caspase-3 pathway increased apoptosis in corpus cavernosum 3) Reduced testosterone levels may be partly responsible for the decreased expression of eNOS and nNOS, and inhibition of NOS/cGMP pathway may be associated with impaired angiogenesis in corpus cavernosum
Hua et al., 2023 (39)	DBP	EMT	RhoA/ROCK	RhoA↑, ROCK 1/2↑, NAP-2↑ TGF-β↑	1) Significantly increased ROS content in HUVECs 2) Activation of the RhoA/ROCK signaling pathway and ROS accumulation in vascular endothelial increased NAP-2 secretion 3) This excess of NAP-2 promoted EMT in urothelial cells through TGF-β pathway
Zhu et al., 2023 (40)	DEHP	EMT	TGF-β/Smad	PFN2↓	1) Decreased EMT levels in urethral tissues 2) Overexpression of PFN2 could activate the Smad/TGF-β1 pathway to upregulate TGF-β1 and promote EMT of epithelial cells
Wu et al., 2024 (41)	DBP	Sex steroids- related pathways Angiogenesis	Androgen signaling	AR↓, TGFB1I1↓	Low levels of TGFB1I1 inhibited AR nuclear translocation and attenuated angiogenesis
Shi et al., 2024 (26)	DEHP	Sex steroids- related pathways	Androgen signaling Estrogen signaling	Srd5α2↓, Cyp11a1↓, Hsd3b↓, Cyp17a1↓, Hsd17b3↓, AR↓, ERβ↑ NOX 1↑, NOX 4↑, MAPK10↑, MAPK14↑	1) Higher levels of testosterone and lower levels of DHT 2) Increased production of ROS and decreased activity of antioxidant enzymes 3) DEHP enhanced ROS production and induced MAPK10 and MAPK14 expression by activating NOX1 and NOX4, subsequently inhibiting Srd5α2 activity

N/A indicates that the included study did not primarily demonstrate a specific mechanism or pathway. ↑ represents the significant increase in the expression of target genes or proteins in the hypospadias group compared to the control group after DBP/DEHP treatment. ↓ indicates that the target genes or proteins are significantly reduced in the hypospadias group.

#### 3.2.1 Sex steroids-related pathways

**Figure 2:**
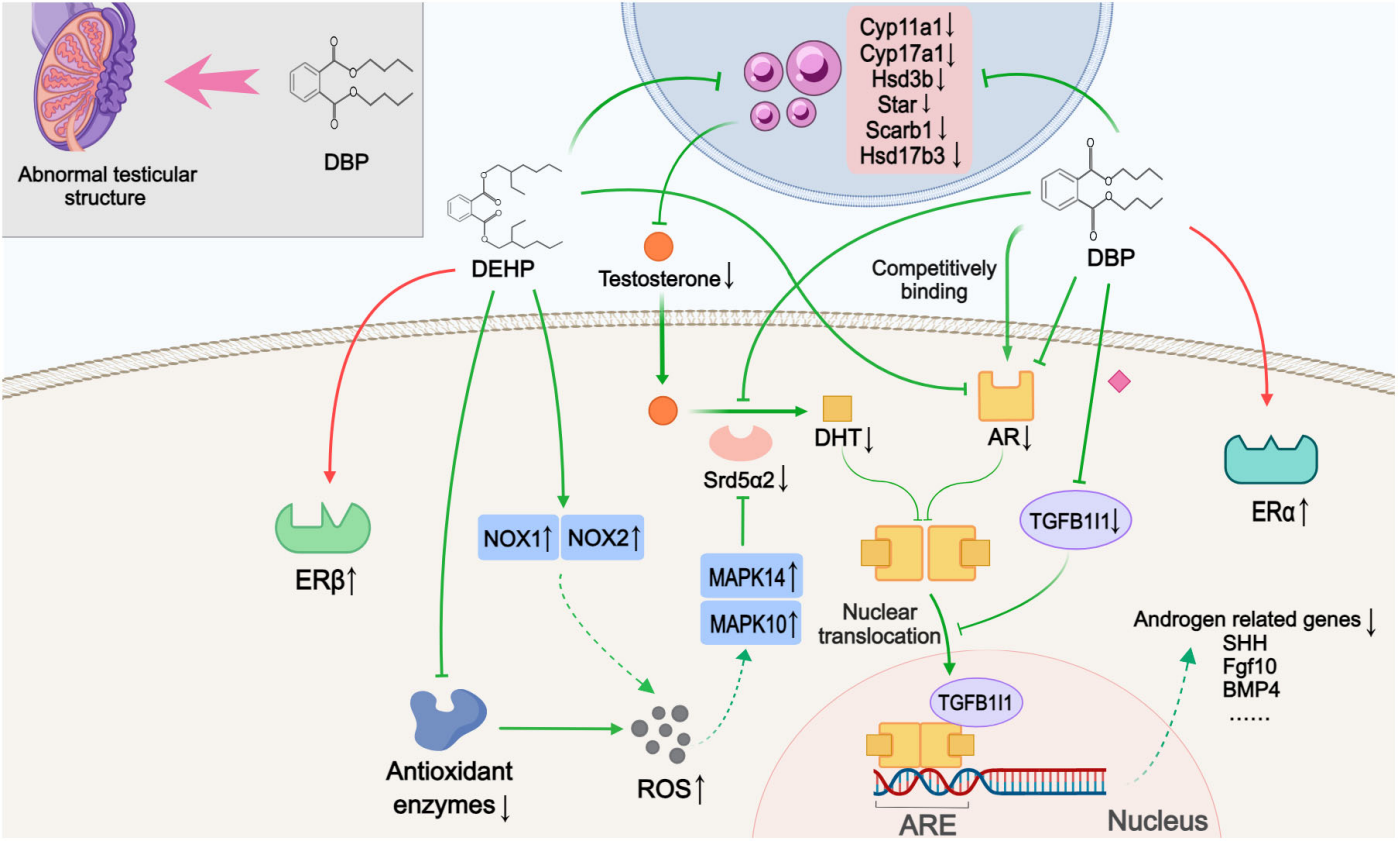
Mechanisms by which phthalates affected sex steroids-related pathways. During the development of offspring, DBP caused abnormal testicular structure. DBP and DEHP hindered testosterone synthesis and androgen signaling through various mechanisms, such as downregulating key enzymes involved in androgen synthesis, antagonizing AR, and reducing TGFB1I1 expression to inhibit nuclear translocation. This impaired androgen pathway may subsequently inhibit the expression of androgen-related genes. Simultaneously, DEHP and DBP also activated estrogen signaling by promoting the expression of ERβ and ERα, respectively. ARE: Androgen response element. (Solid arrows represent mechanisms that have been demonstrated in the included studies). Dashed arrows indicate that mechanisms were proposed but were not experimentally validated in the included studies. Diamond-shaped icons represent the mechanisms that have been validated *in vitro* experiment in the included studies).

Ten studies discussed alterations in sex steroids-related signaling pathways (**Figure 2**). The blocked androgen pathway, as evidenced by the inhibition of testosterone synthesis and impaired androgen receptor (AR) signaling, was observed in the nine included studies. Compared to normal offspring, hypospadias rats exhibited decreased serum testosterone levels and reduced AR expression in the GT. Although one study showed elevated testosterone levels, this was interpreted as a blockage in the conversion of testosterone to dihydrotestosterone (DHT) induced by DEHP (26). Four of these studies further histologically described testicular dysplasia in hypospadias male offspring, including testicular morphological abnormalities, disorganized structure of the seminiferous tubules, aberrant Leydig cell proliferation, and decreased germ cells in adulthood (25, 31, 32, 38). Moreover, three studies found that key enzymes involved in testosterone synthesis, represented by Cyp11a1, Cyp17a1, Hsd3b, Scarb1, Star, Hsd17b3 and Srd5α2, were significantly reduced at the genetic level in DBP- or DEHP-induced hypospadias male fetal rats (26, 31, 32). Among them, Shi et al. (26) indicated that the inhibition of Srd5α2 levels might be caused by DEHP-induced oxidative stress and the subsequent upregulation of MAPK10 and MAPK14. This oxidative stress effect was possibly related to the decreased activity of antioxidant enzymes and the increased expression of NOX1 and NOX4 following DEHP treatment. In addition to the observed decrease in AR expression, two studies investigated the inhibitory effect of DBP at different stages of AR signaling, respectively. Using molecular docking experiments, Zhou et al. (37) showed that DBP had a high affinity for AR and could competitively bind to the androgen receptor to block signal transduction. In the other study, comparing AR levels in the cytoplasm and nucleus, DBP was further found to disrupt AR nuclear translocation by inhibiting TGFB1I1 expression (41). The possible association between the lack of androgen signaling and certain ablated growth factors (Shh, Fgf10, Gli2, Gli3, Bmp4, Wnt5a, Hoxa13, Hoxd13, Fgf8, Fgfr2, etc.) were proposed in three studies (30–32). A limitation was that these studies only detected gene expression levels, in the absence of further experimental validation to confirm that the abnormal expression of these genes is indeed caused by disrupted androgen signaling pathway.

Three studies were categorized as related to estrogenic effects. Interestingly, the data suggested a discrepancy in the selectivity of DBP and DEHP for estrogen receptors (ERs). One study showed that DBP had high affinity values for ERα protein and could bind to and activate the ERα protein (37). Yet another study indicated that in the DEHP-treated groups, with increasing concentrations, DEHP did not seem to have a significant effect on the expression of ERα, but rather promoted the expression of ERβ. In the third study, high expression of the proto-oncogene c-Fos, which was thought to be activated by estrogen signaling, was detected in the GT of hypospadias rats as well as in the human foreskin (36).

#### 3.2.2 EMT

**Figure 3:**
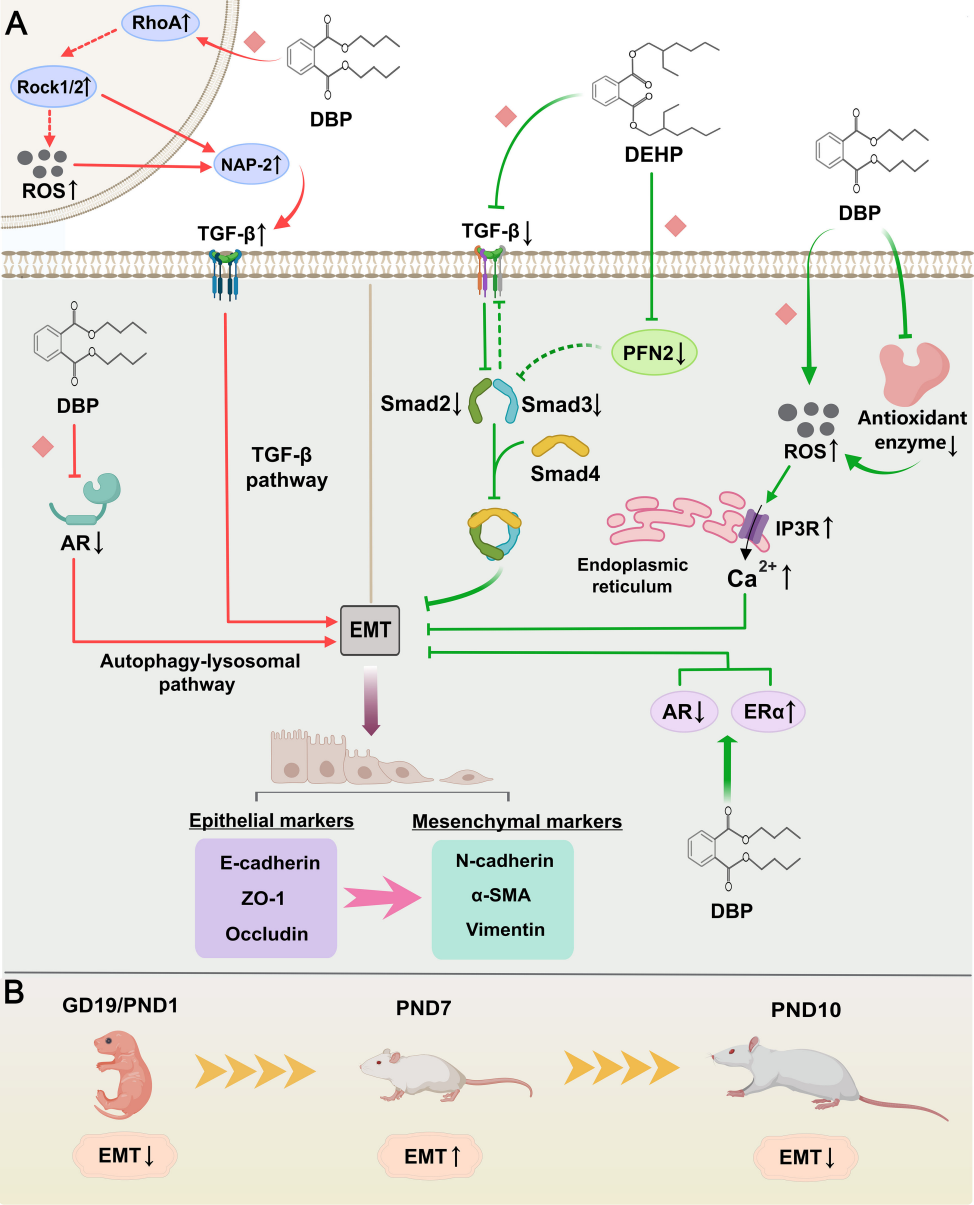
Mechanisms by which phthalates affect epithelial-mesenchymal transition (EMT). **(A)** The green arrows indicate that both DEHP and DBP have inhibitory effects on EMT. DEHP could reduce the expression of the TGF-β/Smad pathway by directly inhibiting or downregulating PFN2, thereby suppressing EMT. Simultaneously, DBP also inhibited EMT not only by upregulating IP3R and intracellular calcium levels, but also by affecting the expression of AR and ERα. The red arrows show that DBP has a promoting effect on EMT. DBP increased TGF-β expression by promoting NAP-2 secretion in the vascular endothelium, which in turn enhanced EMT in urethral epithelial cells. Furthermore, DBP could induce autophagy by impairing androgen signaling and enhance the EMT process through the autophagy-lysosome pathway. **(B)** Changes in EMT levels in phthalate-treated neonatal rats at different sampling times. (Solid arrows represent mechanisms that have been demonstrated in the included studies). Dashed arrows indicate that mechanisms were proposed but were not experimentally validated in the included studies. Diamond-shaped icons represent the mechanisms that have been validated *in vitro* experiment in the included studies).

The six included studies demonstrated that phthalates influence EMT through various mechanisms (**Figure 3A**). Alterations in the expression of epithelial markers (e.g., E-cadherin, ZO-1, occludin) and mesenchymal markers (e.g., N-cadherin, α-SMA, vimentin) were used to evaluate EMT levels. Four studies observed a decreased level of EMT during urethral development in hypospadias offspring. Zhou et al. (27) showed that DEHP treatment inhibited the TGF-β/Smad signaling pathway, contributing to a decline in EMT. In a study by Zhu et al., overexpression of PFN2 was found to promote EMT in urethral epithelial cells through activation of the Smad/TGF-β1 pathway, suggesting that the significantly downregulated PFN2 in hypospadias may inhibit EMT by suppressing the expression of Smad2/3 and TGF-β1 (40). Moreover, a previous study indicated that decreased AR expression and increased ERα expression were potentially associated with decreased EMT (37). One study also found that the oxidative stress effect resulting from the combination of elevated reactive oxygen species (ROS) levels and reduced antioxidant enzyme genes expression after DBP exposure increased IP3R expression and intracellular calcium concentration in urethral epithelial cells, thereby inhibiting EMT (43). However, two other studies reported the contrary results. In a further study of autophagy, Zhao et al. (34) found that DBP-induced autophagy due to impaired androgen signaling could have activated EMT via the autophagy-lysosomal pathway. With the addition of autophagy inhibitors, EMT levels in urethral epithelial cells were also suppressed. Unlike single cell line cultures, another study verified cellular interactions by co-culturing human endothelial and urothelial cells. DBP exposure of vascular endothelium increased NAP-2 secretion via activation of the RhoA/Rock pathway and promotion of ROS production. This vascular endothelial-derived NAP-2 contributed to EMT of urothelial cells by upregulating TGF-β expression (39). Comparing the methodological differences in the above studies, we found that inconsistencies in sampling times resulted in hypospadias pups exhibiting decreased levels of EMT on GD19 or PND1, enhanced EMT on PND7, and again diminished EMT on PND10 (**Figure 3B**). There were four additional studies in relation to the expression of TGF-β1 (23, 24, 28, 38). The issue was that these studies lacked valid evidence to confirm their association with EMT, and therefore, we have not included them in our analysis.

#### 3.2.3 Autophagy

**Figure 4:**
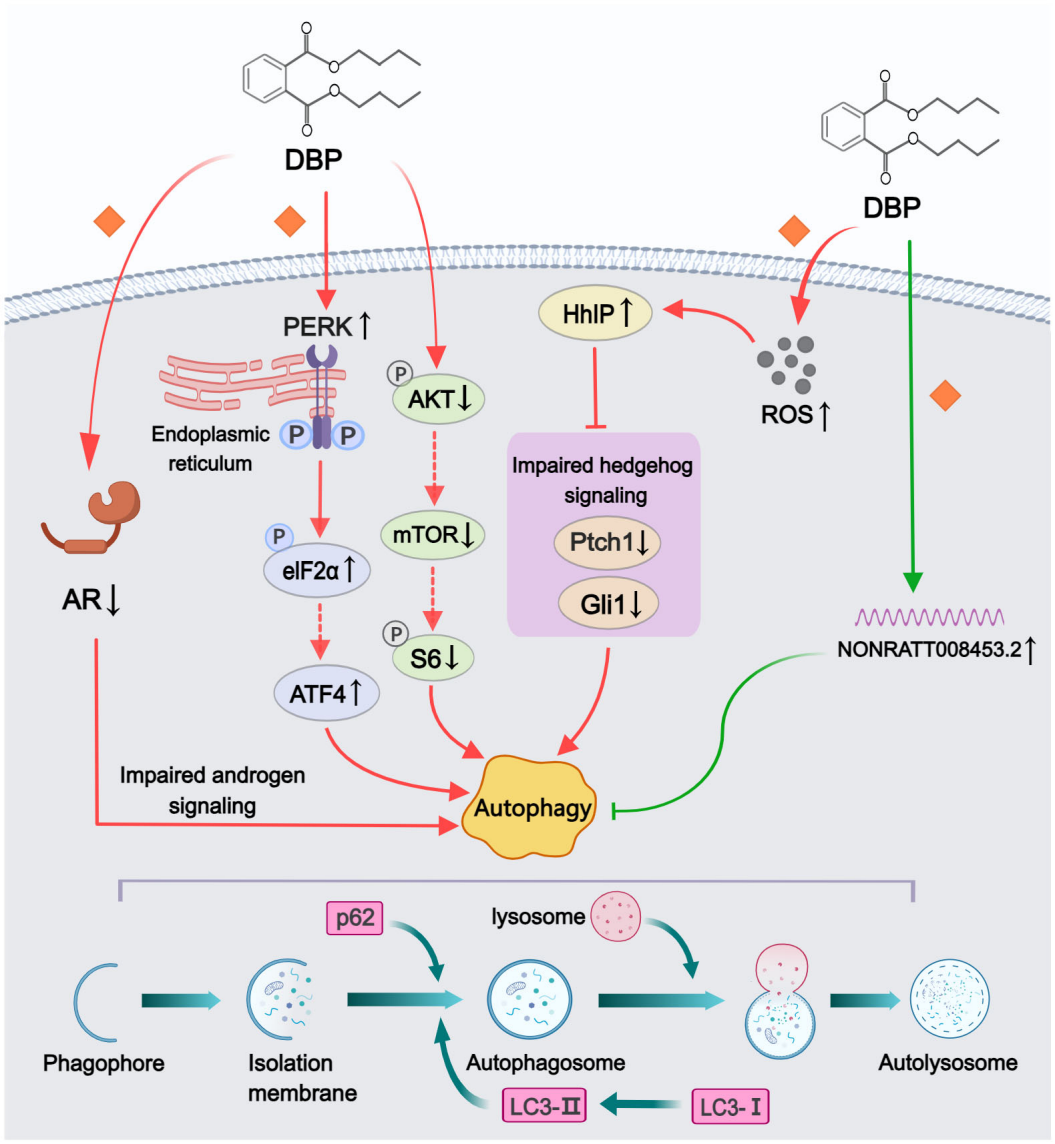
Mechanisms by which phthalates affected autophagy. DBP could enhance autophagy through various pathways, including disrupting androgen signaling, inhibiting the PI3K/Akt/mTOR signaling pathway, impairing the hedgehog signaling pathway, and activating the PERK/eIF2α signaling pathway. However, the elevated levels of NONRATT008453.2 following DBP treatment exhibited autophagy-inhibiting effects. (Solid arrows represent mechanisms that have been demonstrated in the included studies). Dashed arrows indicate that mechanisms were proposed but were not experimentally validated in the included studies. Diamond-shaped icons represent the mechanisms that have been validated *in vitro* experiment in the included studies).

Five studies reported various pathways through which phthalates affect autophagy (**Figure 4**). Among all included studies, DBP was used to construct rat models of hypospadias, and the LC3II/LC3I ratio or polyubiquitin-binding protein p62 were considered crucial indicators for assessing autophagy levels. Three studies documented increased autophagy levels in GTs of hypospadias fetal male rats following maternal exposure to DBP. Using electron microscopy to observe and compare GTs in hypospadias and normal offspring rats, Li et al. (33) found that autophagosomes were only detected in the hypospadias group. Meanwhile, some direct or indirect inhibitory proteins of the autophagy pathway (e.g., p-Akt, p-mTOR and p-S6) were also significantly decreased in the GT of hypospadias fetuses, suggesting that DBP might enhance autophagy by inhibiting the PI3K/Akt/mTOR pathway (33). Another study established a stable AR-transfected urethral epithelial cell line and found that increased AR expression was associated with decreased levels of autophagy compared to the non-transfected group (34). This indicated that DBP-induced impaired androgen signaling also contributed to the promotion of autophagy. In addition, Zhao et al. (44) demonstrated that maternal exposure to DBP activated the PERK/eIF2α pathway, leading to increased autophagy in hypospadias offspring. In transfection experiments of GT fibroblasts *in vitro*, PERK silencing was found to downregulate the phosphorylation of eIF2α and the expression of ATF4, thereby inhibiting autophagy, which inversely confirmed the intrinsic correlation between the PERK/eIF2α pathway and DBP-induced autophagy. The remaining two studies examined the effects of DBP on autophagy levels in cell lines, rather than on the entire GT. DBP exposure has been reported to promote cellular autophagy via inhibition of hedgehog signaling in urethral epithelial cells through upregulation of HhIP expression (42). DBP-induced oxidative stress, through increased HhIP mRNA and protein expression, was also observed to be involved in this regulatory process. However, Feng et al. (35) presented a different view, suggesting that overexpression of NONRATT008453.2 in GT fibroblasts from hypospadias male rat fetuses had an inhibitory effect on cellular autophagy. Although this lncRNA was detected to be highly expressed in GTs of hypospadias rats, direct evidence of autophagy inhibition in GTs was still lacking.

#### 3.2.4 Apoptosis

**Figure 5:**
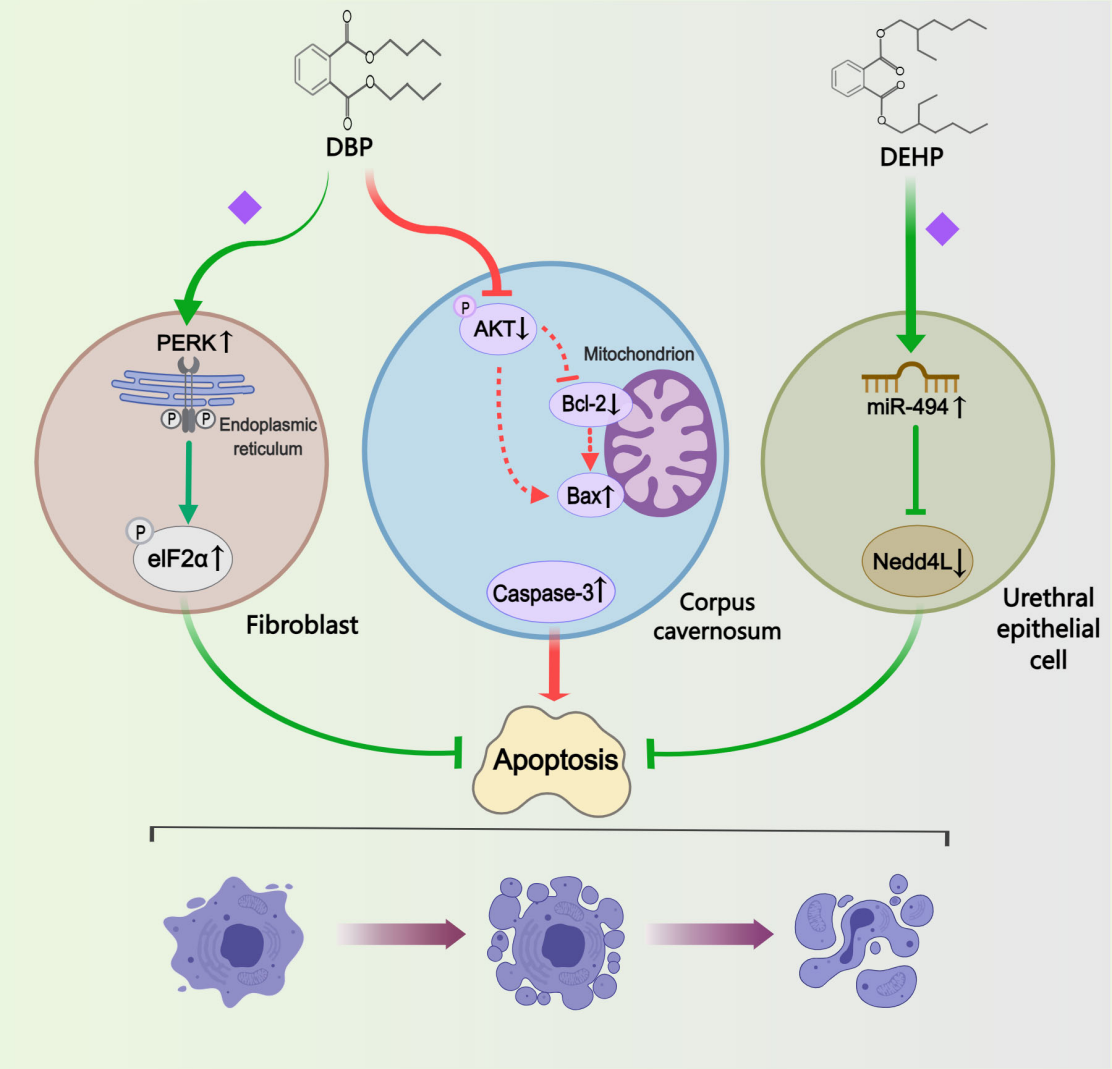
Mechanisms by which phthalates affected apoptosis. In different cells or tissues, the regulation of apoptosis exhibits variability: DBP inhibited fibroblast apoptosis by activating the PERK/eIF2α pathway; DBP enhanced apoptosis in the corpus cavernosum by modulating the Akt/Bax/caspase-3 pathway; and DEHP reduced apoptosis in the urethral epithelial cell by upregulating miR-494. (Solid arrows represent mechanisms that have been demonstrated in the included studies). Dashed arrows indicate that mechanisms were proposed but were not experimentally validated in the included studies. Diamond-shaped icons represent the mechanisms that have been validated *in vitro* experiment in the included studies).

Four studies investigated the regulation of phthalates on cell apoptosis (**Figure 5**). Terminal deoxynucleotidyl transferase dUTP nick end labelling (TUNEL) assay or annexin V-fluorescein isothiocyanate (FITC)/PI double staining were widely adopted to detect cell apoptosis index. In a study on autophagy, Li et al. (33) found inhibition of apoptosis in hypospadias male fetuses, indicating that DBP-induced autophagy may resist apoptosis. The other three studies examined the pathways impacting apoptosis in different tissues or cells. One study analyzed the differential levels of autophagy and apoptosis in GT fibroblasts, suggesting that DBP-induced activation of the PERK/eIF2α signaling pathway and phosphorylation of eIF2α could attenuate apoptosis (44). In addition, Tian et al. (24) demonstrated through *in vitro* transfection experiments that following DEHP treatment, the overexpression of miR-494 and the reduced expression of its target gene Nedd4L can inhibit apoptosis in urethral epithelial cells. Conversely in another study, increased levels of apoptosis were observed in corpus cavernosum of ten-week-old DBP-induced hypospadias offspring rats (38). The Akt/Bax/caspase-3 pathway was implicated in regulating apoptosis levels, with increased caspase-3 protein levels and Bax/Bcl-2 ratios, alongside decreased pAkt/Akt ratios, detected in the hypospadias group.

#### 3.2.5 Angiogenesis

**Figure 6:**
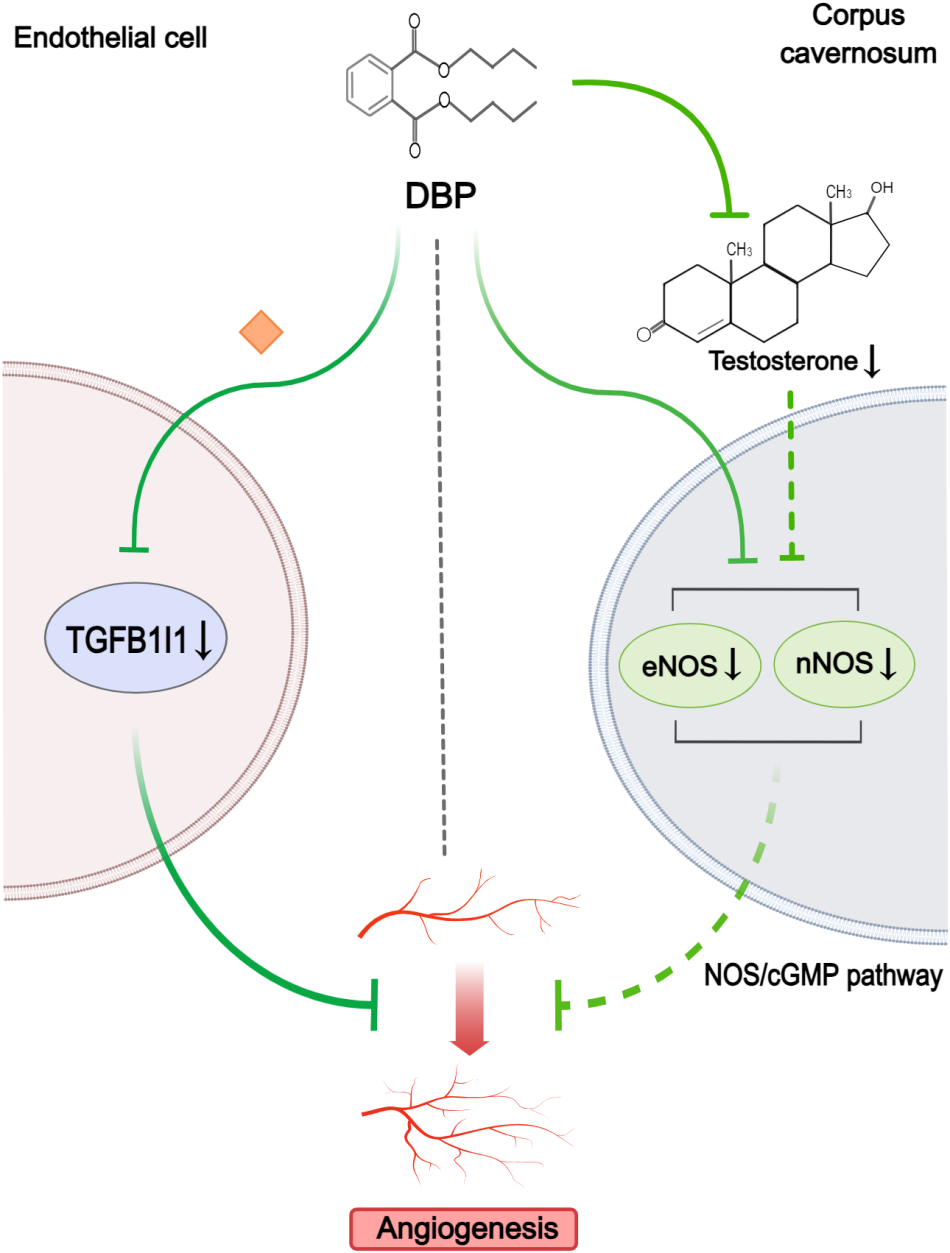
Mechanisms by which phthalates affected angiogenesis. DBP could inhibit angiogenesis by reducing TGFB1I1 expression in endothelial cells. Simultaneously, in the corpus cavernosum, DBP downregulated eNOS and nNOS, with low testosterone levels partly contributing to this reduction, resulting in impaired NOS/cGMP signaling and subsequent angiogenesis disruption. (Solid arrows represent mechanisms that have been demonstrated in the included studies). Dashed arrows indicate that mechanisms were proposed but were not experimentally validated in the included studies. Diamond-shaped icons represent the mechanisms that have been validated *in vitro* experiment in the included studies).

Two studies were considered relevant for angiogenesis (**Figure 6**). Key criteria for evaluating angiogenic potential included tube-forming capacity and endothelial function. One study showed that low expression of TGFB1I1 was involved in DBP-induced aberrant angiogenesis in hypospadias fetal rats (41). By assessing tube formation and endothelial migration *in vitro*, Wu et al. (41) further found that overexpressed TGFB1I1 could mitigate the angiogenic inhibitory effect of DBP exposure. The other study observed only a significant decrease in the expression of eNOS and nNOS in corpus cavernosum of hypospadias rats upon maternal exposure to DBP (38). The author thought the subsequent inhibition of the NOS/cGMP pathway might be associated with depressed angiogenesis and endothelial dysfunction in corpus cavernosum. Additionally, this study suggested that reduced testosterone levels might also be partly responsible for the decreased expression of eNOS and nNOS. However, this study lacked direct assays of angiogenic function.

#### 3.2.6 Interactions among the identified mechanisms

Several studies included in our review have investigated multiple mechanisms, as outlined in the preceding sections. We synthesized the findings from these studies to explore potential interactions among the identified mechanisms. For exploring the correlation between sex steroid signals and EMT, Zhou et al. (37) suggested that the AR downregulation and ERα upregulation induced by DBP may lead to EMT inhibition during urethral development. Observations in hypospadias fetal rats revealed that inhibition of AR expression resulted in an increase in autophagy (34). Notably, low levels of testosterone were associated with the attenuation of the NOS/cGMP pathway, implicating this pathway as one of the means by which androgen signaling influences angiogenesis (38). Considering above results, sex steroids-related signaling pathways might serve as a central regulator among all mechanisms, given its capacity to modulate the remaining ones (**Supplementary Figure S1**).

#### 3.2.7 The mediating role of oxidative stress

Among the included studies, four mentioned that exposure to phthalates during pregnancy promoted the production of ROS in hypospadias fetal rats, and that this increase was dose-dependent (26, 39, 42, 43). And we further notably found that oxidative stress played a crucial role in regulating several identified mechanisms (**Supplementary Figure S2**). For the sex steroids-related pathways, DEHP-induced high levels of ROS interfered with androgen signaling by inhibiting Srd5α2 activity (26). Additionally, oxidative stress has been shown to increase autophagy in urothelial epithelial cells via the ROS-HhIP-Gli-autophagy pathway (42). Moreover, two studies elucidated the opposite effects of oxidative stress on EMT in fetal rats of different developmental stages. In fetal rats on PND1, DBP-induced oxidative stress might inhibit EMT by increasing IP3R expression and intracellular calcium levels in urethral epithelial cells (43). Another study found that oxidative stress in the vascular endothelium after DBP treatment upregulated NAP-2 secretion and subsequently enhanced EMT in urothelial cells on PND7 (39). Consequently, rather than categorizing ROS as a distinct mechanism, we considered it as a significant mediator in the phthalate-induced hypospadias.

## 4 Discussion

Phthalates, extensively used as plasticizers in everyday life, are one of the most prominent EDCs known to impact male urogenital system embryonic development and even adult reproductive health (45). Prenatal exposure to high doses of plasticizers has been identified as a potential risk factor for the development of hypospadias. Numerous animal experimental studies have found direct correlations between phthalates and hypospadias, however, the identified genes or signaling pathways were inconsistent and lacked a holistic overview. Therefore, this review systematically organized existing animal studies on phthalate-induced hypospadias, aiming to construct a more comprehensive understanding of this area. We included a total of 22 studies, all utilizing rodent models (rat or mouse) to establish hypospadias through the application of phthalates. Based on the objectives and the functions of the signaling pathways identified in each study, we have categorized the mechanisms of phthalate-induced hypospadias that have been validated to date into five groups: sex steroids-related signaling pathways, EMT, autophagy, apoptosis, and angiogenesis.

Hypospadias arises from the disruption of sexual differentiation during embryonic development, a process in which sex steroids, especially androgens, play a critical mediating role (46). The presence or absence of androgens during this period determines the ultimate outcome of ambisexual GT development (6). Phthalates are considered to have estrogen-like or anti-androgenic properties due to their ability to mimic estrogen effects in various tissues, including the reproductive system (13). Many studies investigating their pathogenic role in hypospadias have also focused on this aspect. The transmission of androgen signals can be understood as mainly composed of two parts: androgen synthesis and AR pathway. For the first part, testosterone is the major human androgen produced by Leydig cells in the testes (47), however, current research has discovered that phthalates can induce testicular histological structural disturbances, such as disorganized structure of the seminiferous tubules, and abnormal Leydig cell proliferation (31, 32). Additionally, phthalates also downregulate genes involved in androgen synthesis, such as Cyp11a1 and Cyp17a1, and inhibit Srd5a2, which converts testosterone to DHT, a more potent AR ligand (25, 26). The chaotic testicular structure and the suppression of androgen synthase gene expression, both induced by phthalates, collectively contribute to the inhibition of androgen synthesis. For the second part, upon binding and activation by testosterone or DHT, the AR undergoes nuclear translocation. Subsequently, it binds to androgen response elements located on the target DNA, thereby regulating the transcription of specific genes (48). AR expression was found to be markedly suppressed following exposure to phthalates (26, 30). Furthermore, Wu et al. (41) discovered that DBP could alter AR nuclear translocation via inhibiting TGFB1I1 expression. Beyond directly impacting the AR, DBP demonstrated a high affinity for AR proteins, enabling them to bind competitively to AR and exert inhibitory effects (37). In summary, phthalates can impact both androgen synthesis and AR pathway, leading to a reduction in androgen levels and efficacy. The disruption of androgen signaling ultimately led to diminished expression of androgen-related genes like Shh, Fgf10, and their downstream factors (Gli2, Gli3, Bmp4, Wnt5a, Hoxa13, Hoxd13, Fgfr2) (32). It is noteworthy that these genes are key components of the signaling pathways previously mentioned as critical for normal urethral development, and the aberrant expression of certain genes among these has been clinically confirmed to be closely associated with the incidence and severity of hypospadias (49, 50). Moreover, this aberrant sex steroids-related pathway may also suppress the expression of other important genes involved in urethral development. Comparative studies of foreskin tissue from patients with hypospadias and normal children have revealed significant downregulation of Rab25 and Mafb expression in the hypospadias group (51, 52). These genes are closely linked to androgen signal transduction, and their expression is suppressed when androgen signaling is impaired or absent. Phthalates may affect the expression of these genes by inhibiting normal androgen signaling. Contrary to the previous belief that androgens play a dominant role in the embryonic development of male genitalia, current research posits that the coordinated action between androgens and estrogens is key to penile development (53). Phthalates like DBP have been shown to increased ERα protein levels in the GT of hypospadias rats and could also bind to and activate ERα proteins (37). For DEHP, although it appeared to have no effect on the expression of ERα, but instead induced the expression of ERβ (26). To conclude, phthalates may simultaneously disrupt the androgen signaling pathway and anomalously stimulate the estrogen signaling pathway. This dual action can cause an imbalance between these pathways, ultimately culminating in the development of hypospadias.

EMT is an important biological process that allows polarized epithelial cells to lose their typical epithelial characteristics and acquire mesenchymal phenotypes (54). It is indispensable in the development and differentiation of embryonic tissues and organs (55). A critical step in the development of the male urethra in humans is the bilateral fusion of the urethral plates, which subsequently generates the urethral seam and forms a normal tubular urethra (56, 57). EMT, along with apoptosis and cell migration, has been considered as a pivotal cellular process essential for this developmental stage (58). This hypothesis was initially verified by a study detecting several epithelial-mesenchymal co-expression markers associated with EMT in the urethral seam (59). While the promotion of EMT by phthalate exposure has been mentioned in fibrosis (60) and tumor metastasis (61), the potential relationship between the two in hypospadias has not yet been fully elucidated. Among the various EMT regulators, TGF-β has attracted much attention as a major inducer (62, 63). In rat models, gestational exposure to DEHP has been reported to reduce the occurrence of EMT by inhibiting the TGF-β/Smad pathway, resulting in hypospadias (27). Zhu et al. in their research on urinary extracellular vesicles further discovered that the downregulation of TGF-β1 by DEHP may be mediated by PFN2 (40). DBP has also been found to inhibit the EMT process in the GT of hypospadias rats, with ROS and Ca^2+^ potentially playing significant roles in this effect (43). However, two studies observed an enhancement of the EMT process induced by DBP in the GT and urethral epithelial cells of hypospadias rats (34, 39). To explain these seemingly contradictory results, we compared the experimental details of studies focusing on the impact of phthalates on the EMT process in hypospadias. We found that variations in the timing of sample collection could be one contributing factor to the discrepancies observed across different studies. In summary, in studies involving prenatal fetuses or neonatal rats, the EMT process was inhibited by phthalates. By postnatal day seven, phthalates appear to promote EMT, whereas by postnatal day ten, they exhibit an inhibitory effect again. We propose that a plausible explanation for this phenomenon may be attributable to the unique characteristics of penile development in rats. Unlike the human penis, which completes its development prenatally, the rat penis continues to undergo morphological changes after birth (64). Hence, the EMT process in the penile region may continue to undergo dynamic changes after birth in rats, leading to varying results at different sampling times. This suggests that the regulation of EMT by phthalates may not be unidirectional; in other words, it can antagonize the normal rhythmic progression of EMT during development. To elucidate the regulatory mechanisms of phthalates on EMT in penile development, more in-depth explorations are needed, such as conducting longitudinal studies.

Autophagy is the primary intracellular degradation and recycling system that breaks down and recycles aged, damaged, or excess organelles, proteins, and other intracellular components through the lysosomal system (65). During biological growth, autophagy is considered a crucial dynamic metabolic process for maintaining normal cell differentiation and development (66). However, insufficient or excessive autophagy can disrupt intracellular homeostasis, potentially leading to cell death (67). Consequently, abnormal autophagic activity during penile embryonic development may contribute to the formation of hypospadias. Evidence supporting this included the observation of increased autophagosomes and autophagosome-forming proteins (LC3-I and LC3-II) in the GT of rats with DBP-induced hypospadias (33). Li et al. (33) discovered that DBP enhanced autophagy by inhibiting the PI3K/Akt/mTOR signaling pathway. Beyond this classic pathway, Hedgehog pathway has also been implicated in abnormally activated autophagy in hypospadias rats (42). In addition, endoplasmic reticulum stress, a protective cellular stress response, was involved in the regulation of autophagy (68). Activation of endoplasmic reticulum stress induced by DBP has been proved to enhance autophagy through phosphorylation of eIF2α (44). However, NONRATT008453.2, a long non-coding RNA inversely associated with cellular autophagy, was found to be overexpressed under DBP induction in GT fibroblasts from hypospadias rats (35). We propose that a possible explanation is that this overexpression may represent a negative feedback regulatory mechanism by which the organism responds to DBP-induced autophagy in GTs.

Apoptosis is a process of programmed cell death, actively triggered by the cell itself through appropriate means, and it plays a crucial role in maintaining cellular homeostasis in the organism (69). Early research suggested that apoptosis was involved in the morphogenesis and development of GTs, and that abnormal apoptotic function could cause developmental disorders such as hypospadias (70–72). Consistently, measurement of GTs in phthalate-treated hypospadias rats using the TUNEL assay showed a significant decrease in the number of apoptotic cells (33, 44). However, we observed interesting differences in the regulation of apoptosis across various penile tissues induced by phthalates. In urethral epithelial cells, Tian et al. (24) found that miR-494, overexpressed in neonatal hypospadias rats, promoted proliferation and inhibited apoptosis. Upregulation of the PERK/eIF2α signaling pathway has also been reported to attenuate the apoptotic activity of GT fibroblasts in male hypospadias fetal rats (44). Contrary to these findings, Zhou et al. (38) demonstrated that activation of the Akt/Bax/caspase-3 pathway increased apoptosis in corpus cavernosum of 10-week-old hypospadias rats. Furthermore, they confirmed that this is one of the significant causes of erectile dysfunction in rats with hypospadias. Integrating these results, we propose that the regulation of apoptosis by phthalates exhibits tissue-specificity during penile development, and that apoptotic changes varying across different tissues collectively contribute to penile morphological abnormalities and functional impairments. This complex mechanism suggests that merely restoring or inhibiting apoptosis may not adequately counteract the effects of phthalates on penile development.

Angiogenesis is the development of new vascular networks from existing vessels, and its role in embryonic development is increasingly emphasized (73). Both molecular and epidemiological studies have indicated that the penile tissues in children with hypospadias exhibit characteristics of vascular dysfunction (74). In cases of hypospadias, there is abnormal development of the arterial vessels in the foreskin, including the loss of major axial vessels (75). Moreover, penile dartos vessels in hypospadias are distinguished from the normal condition by the presence of small-sized axial ventrolateral vessels and the possible absence of median superior vessels (76). It has been demonstrated that the expression of TGFB1I1 and other pro-angiogenic growth factors was significantly reduced in the GT of DBP-induced hypospadias rats, ultimately resulting in impaired angiogenic capacity (41). Additionally, decreased expression of eNOS and nNOS was observed in DBP-induced hypospadias rats (38). As nitric oxide synthases, eNOS and nNOS exert important vasodilatory effects in normal vascular physiology (77). Meanwhile, endothelial dysfunction was also thought to result from the reduction in eNOS and nNOS (78). The most direct consequence of impaired angiogenesis is the limitation of gas exchange and nutrient supply. However, the specific link between these adverse outcomes and the incidence of hypospadias remains under-researched. We believe that future investigations into this area will enhance our understanding of the relationship between vascular dysfunction and hypospadias.

**Figure 7:**
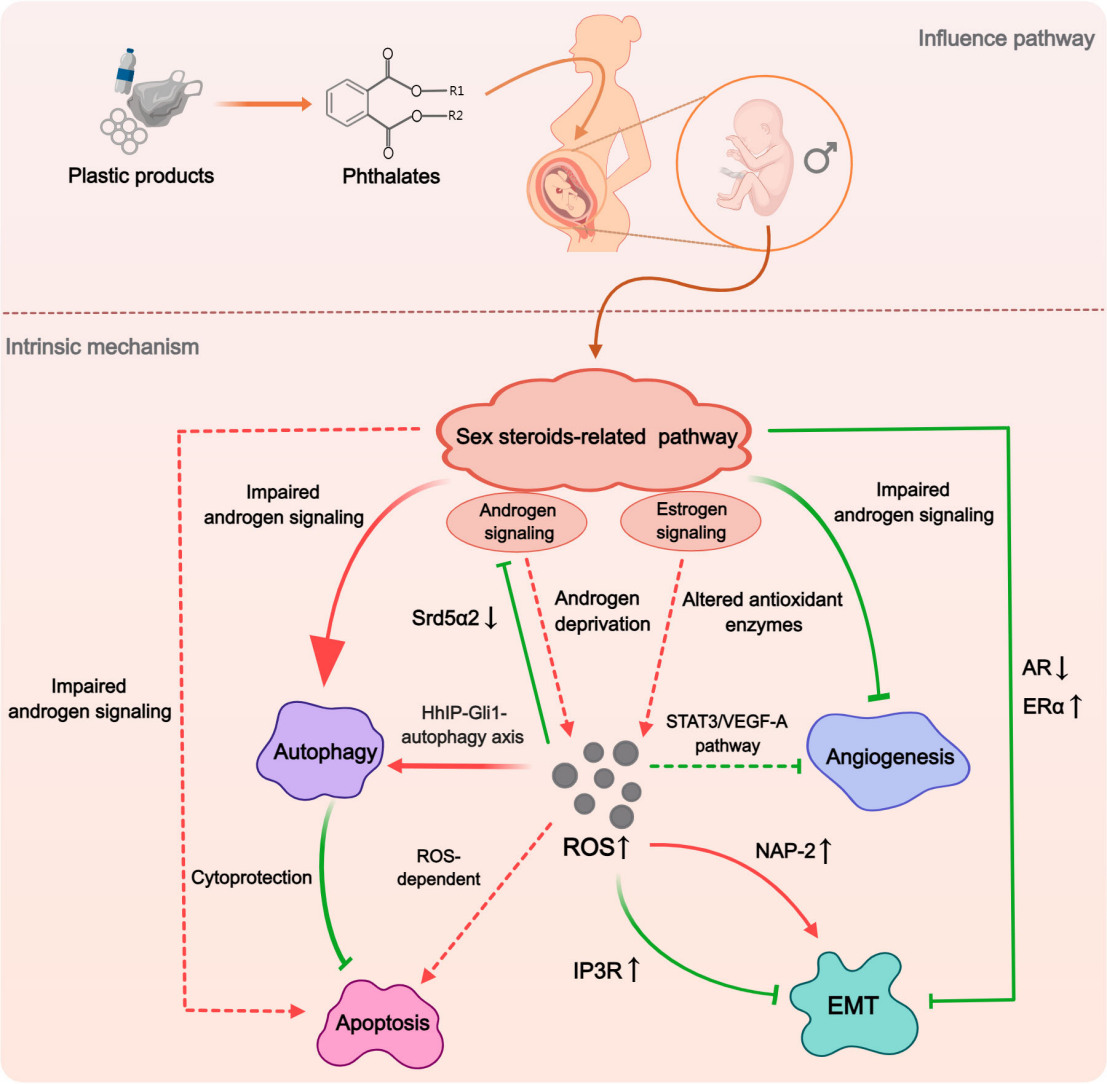
The hypothesized macro-mechanism by which phthalates induce hypospadias. Phthalates would initially impact the sex steroid pathway, which plays the dominant role. The impaired androgen signaling and enhanced estrogen signaling could influence the expression and activity of autophagy, apoptosis, angiogenesis, and EMT. ROS may act as an important mediator in this process, with abnormally elevated ROS participating in the regulation of various mechanisms through different pathways. (Solid arrows represent mechanisms that have been elucidated in the included study). Dashed arrows indicate mechanisms cited from related studies that were not included in this systematic review).

In conducting this systematic review, we have further analyzed multiple mechanisms examined in the included studies. This approach has revealed a significant insight: the five mechanisms identified as potential contributors to the phthalate-induced hypospadias are not isolated from each other, instead, there are intriguing interconnections between them. Of particular interest is the sex steroids-related pathways, as they have the capacity to impact other mechanisms (**Figure 7**). Impaired androgen signaling not only induced enhanced autophagy but also inhibited angiogenesis (34, 38, 41). Furthermore, AR downregulation and ERα upregulation might collectively cause a decrease in EMT (37). This effect could stem from disrupted sex steroids-related pathways impacting the expression of certain genes, such as Rab25, which, as previously mentioned, is closely associated with hypospadias and can regulate EMT through integrin trafficking (79, 80). Although no direct relationship between sex steroids and apoptosis was found in the studies included in this paper, the decline in androgen levels has been shown to promote apoptosis in prostate tissue (81). Additionally, there exists a complex interaction between autophagy and apoptosis. Several studies have found that DBP-induced autophagy can inhibit apoptotic activity to some extent (33, 44). The possible reason for this lied in the pro-survival mechanism of autophagy, which may alleviate endoplasmic reticulum load and resist apoptosis in normal cells (82, 83). On the other hand, over-activated autophagy has also been suggested to play a cytotoxic role in conjunction with apoptosis (84). Therefore, abnormal sex steroids levels may also potentially influence the level of apoptosis during penile development through autophagy. In summary, while phthalates pose a threat to multiple functions during penile development, the sex steroids-related pathways appear to be central, potentially acting as the primary regulatory factor for other functional alterations.

ROS is a generic term for a class of unstable oxygen-containing chemicals associated with oxygen metabolism (85). They play a complex role in organisms, not only causing oxidative damage to cells or tissues, but also acting as signaling molecules (86). The ability of phthalates to increase ROS production and inhibit antioxidant enzyme activity has been demonstrated in numerous experiments (87, 88). Through literature review, we found that ROS may serve as a critical mediator in the phthalate-induced hypospadias (**Figure 7**). In the studies included in this review, ROS has been closely associated with impaired androgen signaling pathways, upregulation of autophagy, and disruption of the EMT process (26, 39, 42, 43). Although no studies have specifically explored the association between ROS and either apoptosis or angiogenesis in phthalate-induced hypospadias, oxidative stress has been demonstrated to induce cellular apoptosis through both mitochondria-dependent and mitochondria-independent pathways across various cell types (89). Regarding angiogenesis, low concentrations of ROS in cells were considered as promoting factors for angiogenesis, however, high levels of ROS in mitochondria could inhibit the STAT3/VEGF-A pathway to reduce angiogenesis (90, 91). Interestingly, the sex steroids-related pathways may not only serve as the initiating factor for other mechanisms, as previously hypothesized, but can also be a crucial route by which phthalates increase ROS production. On the one hand, in a study of cardiomyocytes, testosterone deficiency was found to induce oxidative stress (92). On the other hand, estrogen signaling appeared to exert pro-oxidant effects, with ER potentially serving as the upstream signal that promotes ROS generation (93, 94).

Based on our findings, we propose a plausible macroscopic mechanism by which phthalates induce hypospadias. When phthalates enter the fetal body during the period of sexual differentiation, they would exert estrogen-like properties. They initially activate the estrogen signaling pathway while inhibiting the androgen signaling pathway. This alteration in sex steroids-related pathways stimulates the production of ROS in the GT. Elevated ROS levels further suppress the androgen signaling pathway during genital development and promote cellular autophagy. The impact on cellular apoptosis is tissue-specific; in epithelial tissues, increased autophagy exerts a cytoprotective effect inhibiting apoptosis, whereas in corpus spongiosum tissues, the pro-apoptotic effects of ROS may predominate, leading to increased cell death. Meanwhile, ROS also activates signaling pathways that antagonize the normal process of EMT and inhibit angiogenesis during penile development. This multifunctional abnormality during penile development, triggered by sex steroids-related pathways with ROS acting as the mediator, ultimately leads to the occurrence of hypospadias. We propose that this comprehensive perspective offers novel insights into the prevention of hypospadias induced by phthalates. Firstly, the most critical aspect is to restore the balance in the sex steroids levels disrupted by phthalates, as alterations in sex steroids-related pathways are the primary initiators of other functional abnormalities associated with hypospadias. Designing intervention strategies focused on the estrogen-like properties of phthalates is likely the most fundamental approach. Simultaneously, another potentially more feasible key strategy involves reducing the production of ROS induced by phthalates, since elevated ROS levels are crucial mediators of various functional abnormalities caused by phthalates. It is noteworthy that maternal intake of certain antioxidants, such as folic acid and vitamins, during pregnancy has been shown in retrospective studies and animal research to reduce the incidence of hypospadias (95, 96). However, larger-scale clinical studies are still needed to substantiate the preventive effects of antioxidants on hypospadias.

Secondly, based on the findings of our literature review, we recommend that future animal experiments on phthalate-induced hypospadias should place greater emphasis on the timing of sample collection. On the one hand, studies have demonstrated that the effects of phthalates on EMT during penile development vary depending on the timing of tissue sampling. This difference might be attributed to the continued postnatal development of the penis in rats, but it also implies that the influence of phthalates may be time-specific. On the other hand, we discovered that phthalates may regulate apoptosis in cells from different penile tissues with tissue specificity. If sampling is done during embryonic development, the various penile tissues in rats have not yet fully developed and are difficult to distinguish, making it easy to overlook tissue specificity in experimental results. However, if samples are collected after birth, although penile tissues have matured, since embryonic development has already concluded, we may only detect the biological abnormalities caused by phthalates, missing potential early signaling pathway changes. In summary, different sampling times each have their advantages and disadvantages. Therefore, incorporating multiple sampling points into a single study for a temporal analysis may yield significant new insights into the mechanisms by which phthalates lead to hypospadias.

Hypospadias is also considered a phenotype of Disorders of Sexual Development (DSD). DSD encompasses a range of conditions characterized by abnormal sexual differentiation, which in males, can also present as cryptorchidism and micropenis (97). Our further investigation of the existing literature reveals a significant positive correlation between phthalate exposure and the incidence of cryptorchidism, likely due to disruptions in estrogen and androgen signaling pathways (37). Additionally, ROS play a critical role; studies indicate that DEHP can cause abnormal testicular development by altering oxidative balance and modulating SIRT1/PGC1α levels (98). This testicular toxicity can be mitigated by antioxidants such as vanillic acid and vitamin C (99). Thus, the reproductive toxicity of phthalates may not be limited to the induction of hypospadias but may also exert an inductive effect on other manifestations of DSD. Research on phthalates’ role in causing micropenis is relatively scarce, which could serve as a potential direction for future studies.

## 5 Limitations

It is necessary to point out here the limitations of this review caused by the characteristics of the included studies. Firstly, although the keyword “phthalates” was used in the literature search, only two types of phthalates, DEHP and DBP, were employed in all the studies. Thus, the applicability of these findings to other types of phthalates requires further experimental validation. Secondly, since current *in vivo* experiments utilize rodent models, caution is needed when applying the conclusions of this article to explain the mechanisms by which phthalates induce hypospadias in humans, considering the species-specific differences in embryonic development.

Finally, this review has discussed only the mechanisms by which individual phthalates induce hypospadias; however, in real-world conditions, humans are often exposed to multiple EDCs (100). Other EDCs may also disrupt the pathways mentioned here, interfering with normal cellular activities. For example, finasteride can downregulate apoptosis levels (101), and vinclozolin can unbalance sex steroids-related pathways (102, 103). Therefore, concurrent exposure to phthalates and various other EDCs during pregnancy may further amplify the disruptions of the mechanisms discussed in this review, potentially increasing the risk of hypospadias, although additional research is needed to substantiate this.

## 6 Conclusion

This systematic review dissects the intricate mechanisms underlying phthalate-induced hypospadias revealed through rodent model studies, organizing them into five distinct categories: sex steroids-related pathways, EMT, autophagy, apoptosis, and angiogenesis. We find that phthalates may initially disrupt the balance of sex steroids-related pathways, leading to abnormally elevated levels of ROS. This escalation in ROS subsequently precipitates other functional abnormalities, ultimately resulting in the development of hypospadias. Our findings suggest that interventions such as antioxidant supplementation during pregnancy may mitigate the teratogenic effects of phthalates. Future research should expand on the types of phthalates studied and conduct longitudinal studies to enhance our understanding for phthalate-induced hypospadias.

## Data Availability

The original contributions presented in the study are included in the article/**Supplementary Material.** Further inquiries can be directed to the corresponding author.

## Glossary

EDCs: Endocrine disrupting chemicals
DSD: Disorders of Sex Development
DEHP: Di-(2-ethylhexyl) phthalate
MEHP: Mono-(2-ethylhexyl) phthalate
DBP: Di-n-butyl phthalate
MBP: Mono-butyl phthalate
GT: Genital tubercle
BW: Body Weight
GD: Gestational day
PND: Postnatal day
PUECs: Primary urethral epithelial cells
HUVECs: Human umbilical vein endothelial cells
LncRNA: Long Non-Coding RNA
ROS: Reactive oxygen species
EMT: Epithelial-mesenchymal transition
α-SMA: Alpha-Smooth Muscle Actin
ZO-1: Zonula Occludens-1
AR: Androgen receptor
ER: Estrogen Receptor
DHT: Dihydrotestosterone
Shh: Sonic hedgehog
Ptch1: Patched 1
Bmp4/7: Bone morphogenetic protein 4/7
Fgf8/10: Fibroblast growth factor 8/10
Fgfr2: Fibroblast growth factor receptor 2
Srd5a2: Steroid 5 Alpha-Reductase 2
Hsd17b3: Hydroxysteroid 17-Beta Dehydrogenase 3
Hsd3b: Hydroxysteroid 3-Beta Dehydrogenase
Scarb1: Scavenger Receptor Class B Member 1
Star: Steroidogenic Acute Regulatory Protein
ATF4: Activating Transcription Factor 4
PFN2: Profilin 2
IP3R: Inositol 1,4,5-Trisphosphate Receptor
c-Fos: Cellular Proto-Oncogene Fos
FITC: Fluorescein isothiocyanate
TUNEL: Terminal deoxynucleotidyl transferase dUTP nick end labelling
LC3-I/II: Microtubule-Associated Protein 1 Light Chain 3 Alpha/Beta
miR-494: MicroRNA-494
Nedd4L: NEDD4 Like E3 Ubiquitin Protein Ligase
HhIP: Hedgehog Interacting Protein
Gli1/2/3: Glioma-Associated Oncogene Family Zinc Finger 1/2/3
Bcl-2: B-Cell Lymphoma 2
Bax: Bcl-2-Associated X Protein
Caspase-3: Cysteine-aspartic Acid Protease 3
eNOS: Endothelial Nitric Oxide Synthase
nNOS: Neuronal Nitric Oxide Synthase
cGMP: Cyclic Guanosine Monophosphate
RhoA: Ras Homolog Family Member A
ROCK 1/2: Rho-Associated Coiled-Coil Containing Protein Kinase 1/2
NAP-2: Neutrophil-Activating Protein 2
NOX 1/4: NADPH Oxidase 1/4
MAPK10/14: Mitogen-Activated Protein Kinase 10/14
GSK-3β: Glycogen Synthase Kinase 3 Beta
NFκB: Nuclear Factor Kappa B
Wnt5a: Wingless-Type MMTV Integration Site Family, Member 5A
Hoxa13: Homeobox A13
Hoxd13: Homeobox D13
Rab25: Ras-Related Protein Rab-25
Mafb: MAF BZIP Transcription Factor B
TGF-βrIII: Transforming growth factor beta receptor III
TGF-β1: Transforming growth factor beta 1
TGFB1I1: Transforming Growth Factor Beta 1 Induced Transcript 1
Smad2/3: Mothers Against Decapentaplegic Homolog 2/3
Cyp11a1: Cytochrome P450 Family 11 Subfamily A Member 1
Cyp17a1: Cytochrome P450 Family 17 Subfamily A Member 1
PI3K: Phosphoinositide 3-Kinase
STAT3: Signal Transducer and Activator of Transcription 3
VEGF-A: Vascular Endothelial Growth Factor A
p-Akt: Phosphorylated Protein Kinase B
p-S6: Phosphorylated Ribosomal Protein S6
p-mTOR: Phosphorylated Mechanistic Target of Rapamycin
p-eIF2α: Phosphorylated Eukaryotic Initiation Factor 2 Alpha
p-PERK: Phosphorylated Protein Kinase R-like Endoplasmic Reticulum Kinase
