# Supplementary material for: The potential mechanisms underlying phthalate-induced hypospadias: a systematic review of rodent model studies
Source: Front Endocrinol (Lausanne). 2024 Dec 4;15:1490011. doi: 10.3389/fendo.2024.1490011 (PMC11652206; doi:10.3389/fendo.2024.1490011)
Supplement: Supplementary file 1 [file DataSheet1.docx]

Supplementary Material

# Supplementary **Table S1. Search strategy.**

| - **Pubmed:**   ("phthalates"[All Fields] OR "phthalic acid"[Supplementary Concept] OR "phthalic acid"[All Fields] OR "phthalate"[All Fields]) AND ("hypospadias"[MeSH Terms] OR "hypospadias"[All Fields] OR "hypospadia"[All Fields]) |
| --- |
| - **Web of science:**   ALL=("phthalates" OR "phthalic acid" OR "phthalate") AND ALL=(hypospadias) |

# Supplementary Figures


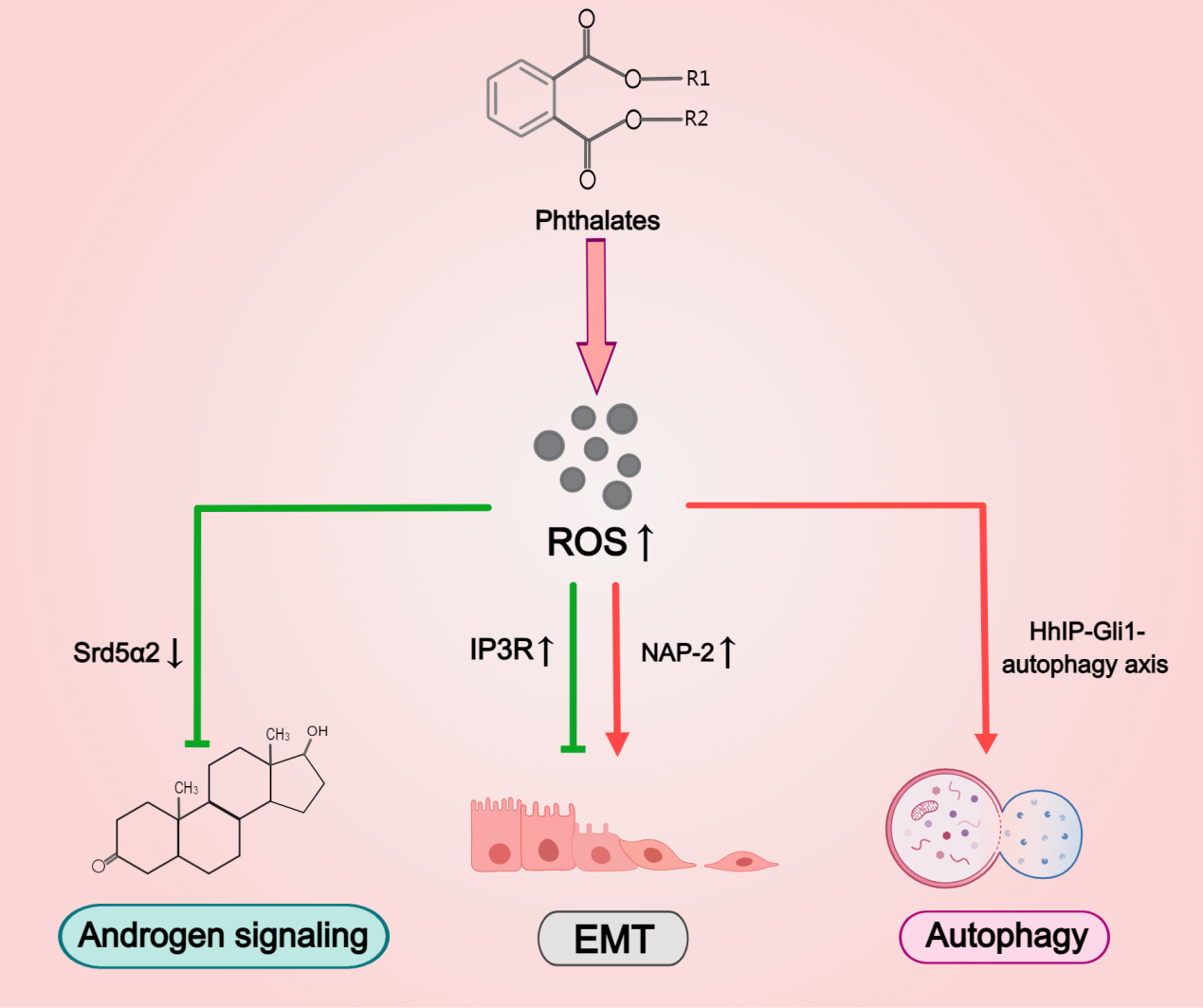


**Supplementary Figure S1.** The crucial mediating role of ROS in phthalate-induced hypospadias. DEHP inhibited androgen signaling by inducing ROS production, which suppresses Srd5α2 activity. In addition, DBP-induced oxidative stress could enhance autophagy via the ROS-HhIP-Gli-autophagy axis. Elevated ROS levels following DBP treatment had opposite effects on EMT at different stages of penis development. Increased ROS not only suppressed EMT by upregulating IP3R expression but also could promote EMT by increasing the secretion of vascular endothelial NAP-2.


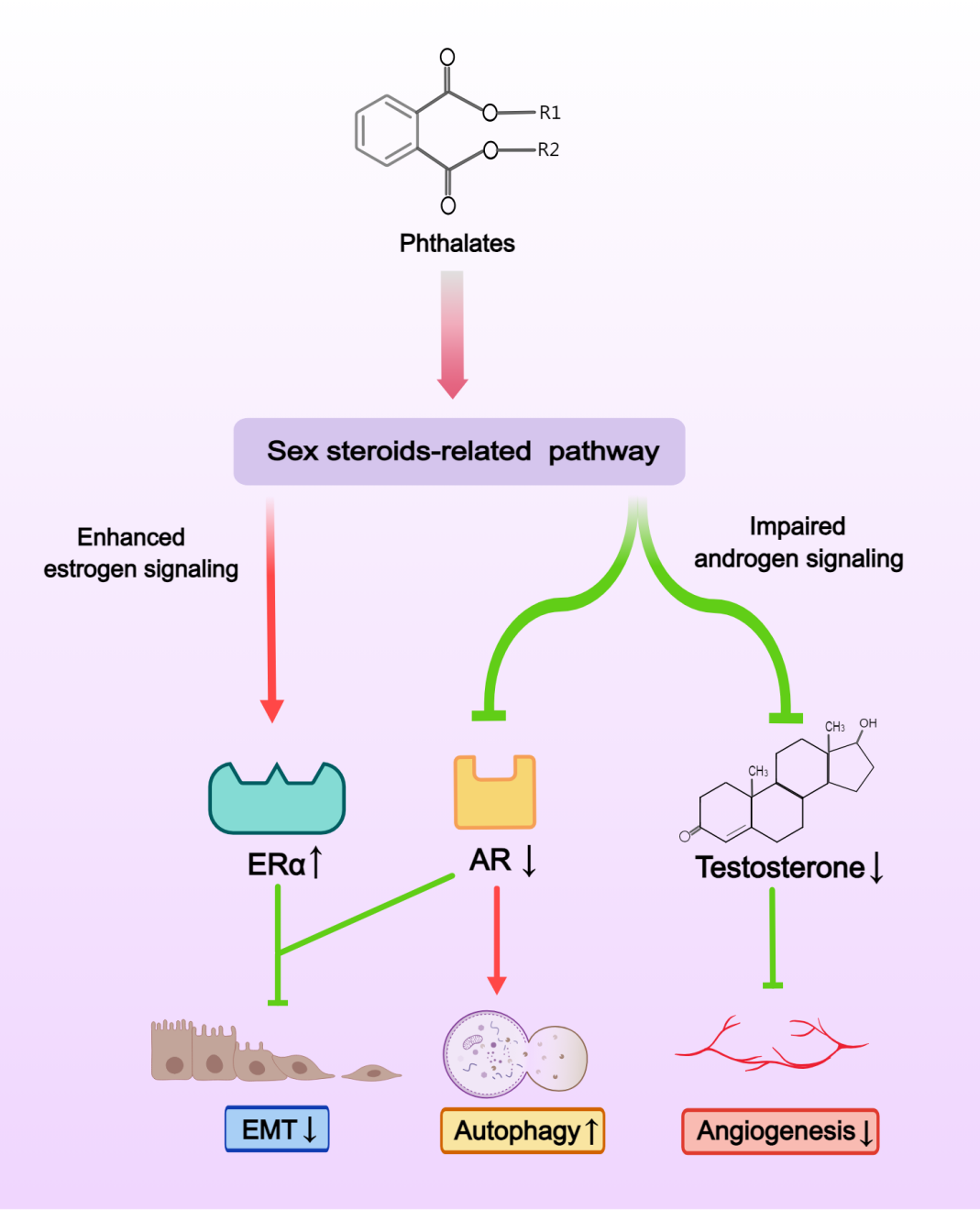


**Supplementary Figure S2.** The core regulatory role of the sex steroids-related pathways in phthalate-induced hypospadias. DBP impaired androgen signaling to inhibit angiogenesis and promote cellular autophagy. Additionally, the downregulation of AR signaling combined with the upregulation of ER signaling collectively led to the suppression of EMT process.
